# Responses of Soil Microbial Communities to Experimental Warming in Alpine Grasslands on the Qinghai-Tibet Plateau

**DOI:** 10.1371/journal.pone.0103859

**Published:** 2014-08-01

**Authors:** Bin Zhang, Shengyun Chen, Xingyuan He, Wenjie Liu, Qian Zhao, Lin Zhao, Chunjie Tian

**Affiliations:** 1 Northeast Institute of Geography and Agroecology, Chinese Academy of Sciences, Changchun, China; 2 State Key Laboratory of Cryospheric Sciences, Cold and Arid Regions Environmental and Engineering Research Institute, Chinese Academy of Sciences, Lanzhou, China; 3 Observation and Research Station of Qinghai-Tibet Plateau, Cold and Arid Regions Environmental and Engineering Research Institute, Chinese Academy of Sciences, Golmud, China; Institute of Tibetan Plateau Research, China

## Abstract

Global surface temperature is predicted to increase by at least 1.5°C by the end of this century. However, the response of soil microbial communities to global warming is still poorly understood, especially in high-elevation grasslands. We therefore conducted an experiment on three types of alpine grasslands on the Qinghai-Tibet Plateau to study the effect of experimental warming on abundance and composition of soil microbial communities at 0–10 and 10–20 cm depths. Plots were passively warmed for 3 years using open-top chambers and compared to adjacent control plots at ambient temperature. Soil microbial communities were assessed using phospholipid fatty acid (PLFA) analysis. We found that 3 years of experimental warming consistently and significantly increased microbial biomass at the 0–10 cm soil depth of alpine swamp meadow (ASM) and alpine steppe (AS) grasslands, and at both the 0–10 and 10–20 cm soil depths of alpine meadow (AM) grasslands, due primarily to the changes in soil temperature, moisture, and plant coverage. Soil microbial community composition was also significantly affected by warming at the 0–10 cm soil depth of ASM and AM and at the 10–20 cm soil depth of AM. Warming significantly decreased the ratio of fungi to bacteria and thus induced a community shift towards bacteria at the 0–10 cm soil depth of ASM and AM. While the ratio of arbuscular mycorrhizal fungi to saprotrophic fungi (AMF/SF) was significantly decreased by warming at the 0–10 cm soil depth of ASM, it was increased at the 0–10 cm soil depth of AM. These results indicate that warming had a strong influence on soil microbial communities in the studied high-elevation grasslands and that the effect was dependent on grassland type.

## Introduction

The global average surface temperature has increased by 0.85°C since 1880, due primarily to an anthropogenic increase in greenhouse gas concentrations, and is predicted to increase by at least 1.5°C by the end of the 21^st^ century [1]. These temperature increases could have profound effects on biodiversity and ecosystem functioning [2], [3], which subsequently influence feedback loops between ecosystem carbon (C) storage and climate warming [4]. While climate-modeling studies predict positive feedback from warming in the form of decreased C storage due to soil respiration [5]–[7], experimental warming studies on ecosystem C storage give controversial results [8], [9]. Such controversy stems partially from uncertainties regarding the response of belowground microbial communities to climate warming as soil microorganisms play crucial roles in regulating ecosystem functioning and soil biogeochemistry [4], [10], [11]. Therefore, a better understanding of microbial responses to elevated temperature is critical to predicting the effects of future climate warming.

Experimental warming with a modest increase in soil temperature has been shown to influence the biomass and composition of soil microbial communities. While some warming studies showed a decrease in soil microbial biomass when exposed to elevated temperature [12]–[16], others reported an increase [4], [9], [17], [18]. The picture was more complex with regards to the warming effect on the composition of soil microbial communities. For example, Zhang et al. [15] found that warming increased the presence of fungi in the soil of unclipped tallgrass prairie as evidenced by both microbial substrate utilization patterns and the profiles of phospholipid fatty acids (PLFAs). Deslippe et al. [12] noted a significant increase in the evenness of fungal communities in warmed Arctic tundra. However, it was also reported that warming decreased the relative abundance of fungi in a northeastern forest [13] and a subarctic heath ecosystem [14]. Furthermore, study by Frey et al. [13] indicated that the soil microbial community shifted towards gram-positive (G+) bacteria and actinomycetes after 12 years of warming. These changes suggest that the warming effects on biomass and composition of soil microbial communities are linked to a wider range of factors than temperature alone. In addition, warming studies are generally restricted to low-elevation areas, and less attention has been paid to the warming effect on soil microorganisms in high-elevation grasslands. Soils in high-elevation grasslands are exposed to harsher environmental conditions as a result of a colder climate and less favorable nutrient conditions [19], [20], which may induce different responses by microbial communities to warming when compared with those in low-elevation areas.

In this study, we examined the impacts of experimental warming on abundance and composition of soil microbial communities in alpine grasslands on the Qinghai-Tibet Plateau. The Qinghai-Tibet Plateau is considered the “Third Pole” of the earth and has experienced a striking warming trend over the last half-century [21], [22], providing a unique opportunity to explore the effects of warming in high-elevation grasslands. Three types of alpine grasslands (alpine swamp meadow, alpine meadow, and alpine steppe) were analyzed to determine whether the response of soil microbial communities to warming depended on grassland type. Soil microbial communities were characterized by PLFA analysis as it is a sensitive tool for measuring microbial biomass and fingerprinting microbial community composition [23], [24].

## Materials and Methods

### Site Description and Experimental Setup

The study site is located near the Beiluhe Observation and Research Station (34°51′ N, 92°56′ E, Altitude: 4659 m), Cold and Arid Regions Environmental and Engineering Research Institute, Chinese Academy of Sciences. This region is typical of the continental climate of the plateau and is characterized by rarefied air, low air temperature, little but concentrated rainfall, low pressure, and strong ultraviolet radiation [25]. The mean annual air temperature is −3.8°C and the mean annual precipitation is 383 mm [26]. Precipitation falls mainly during the summer monsoon season and the freeze period lasts from September to April. The soils are classified as Calcic Kastanozem and Gelic Arenosols in the WRB soil classification system [27], [28]. The three most typical alpine grasslands near this site are alpine swamp meadow (ASM), alpine meadow (AM), and alpine steppe (AS). ASM is populated by hardy perennial hygrophilous herbs, principally *Kobresia tibetica* and *Carex moorcroftii*, which thrive under the waterlogged or moist soil conditions. AM is characterized by cold meso-perennial herbs growing under soil conditions with moderate water availability and consists mainly of *K. pygmaea*, *K. robusta*, and *Androsace tapete*. AS is dominated by hardy perennial xeric herbs and dwarf shrubs such as *Stipa purperea*, *Carex moorcroftii*, and *Saussurea arenaria*.

An area of 30 m×30 m was fenced off in each type of alpine grassland to protect against grazing. Permission to conduct experiment here was issued by the Cold and Arid Regions Environmental and Engineering Research Institute, Chinese Academy of Sciences. In fall 2008, three replicated warming plots were set up within the fenced area using open-top chambers (OTCs) as a passive warming device, modeling the approach used in the International Tundra Experiment (ITEX) [29], [30]. Parallel control plots were established 2 m apart from their adjacent warming plots. The 1 mm-thick fiberglass OTCs, each 40 cm tall, are shaped like truncated cones with inwardly inclined sides (60° with respect to the horizontal) and a 1.48 m bottom diameter (Sun-Lite HP, Solar Components Corporation, Manchester, USA). These OTCs remained on the warming plots throughout the experimental period.

### Soil Sampling and Analysis

On October 11^th^, 2011, seven randomized soil cores (3.14 cm in diameter) were collected per plot from a depth of 0 to 20 cm using a hand auger. The soil cores were cut into two segments: 0–10 cm and 10–20 cm. Segments from the same plot were separated on the basis of depth, placed in plastic bags, and kept cool until processed in the laboratory. After removal of visible fresh roots and plant materials, the soils were homogenized, passed through a 2 mm sieve, and kept in a refrigerator at 4°C. One sub-sample was air-dried for analysis of soil properties and another was freeze-dried for PLFA extraction.

Organic carbon (C) and total nitrogen (N) in the soil were determined by dichromate oxidation using the Walkley-Black procedure [31] and the micro-Kjeldhal procedure [32], respectively. Soil pH was measured in a 1∶5 soil/water suspension. Soil moisture was calculated from weight loss during oven drying at 105°C for 24 h. Soil temperature at the 0, 10, and 20 cm depths were recorded year-round on data-loggers (CR1000, Campbell Co., Ltd.) at half hour intervals by automatic thermal sensors.

### Phospholipid Fatty Acid Analysis

PLFA extraction was conducted following the procedure described by Bossio and Scow [33]. Briefly, lipids were extracted in a single-phase chloroform-methanol- citrate buffer system. Phospholipids were separated from neutral lipids and glycolipids on solid phase extraction columns (Supelco, Inc., Bellefonte, PA). After methylation of the polar lipids, PLFA methyl esters were analyzed by an Agilent 6850N gas chromatograph (GC, Agilent Tech. Co., USA) equipped with an HP-5 capillary column (30 m×0.32 mm×0.25 µm) and a flame ionization detector (FID). The MIDI Sherlock Microbial Identification System (Microbial ID Inc., Newark, USA) was used to identify fatty acids. Nonadecanoic acid methyl ester (19:0, Sigma) was added as an internal standard and used to convert fatty acid peak areas to absolute abundance.

A total of 53 different PLFAs (C chain length of 12 to 20) were detected in this study. Thirty-six individual PLFAs, accounting for >98% of the total in mole percentages, were consistently present in all samples and therefore used for data analysis. The sum of all PLFAs indicates the total microbial biomass. We used the sum of i14:0, a15:0, i15:0, i16:0, a17:0, and i17:0 to determine Gm+ bacteria and the sum of 16:1 2OH, 16:1ω7c, 16:1ω9c, cy17:0, 17:1ω8c, 18:1ω7c, and cy19:0 to determine gram-negative bacteria (Gm-) [34], [35]. The sum of 10Me16:0, 10Me17:0, and 10Me18:0 was used to ascertain total biomass of actinomycetes. We used 16:1ω5c to determine the amount of arbuscular mycorrhizal fungi (AMF), the sum of 18:1ω9c and 18:2ω6c to determine the amount of saprotrophic fungi (SF), 20:4ω6,9,12,15c to determine the amount of protozoa, and the sum of 16:1ω5c, 18:1ω9c, 18:2ω6c to determine the amount of fungi [36]. Ratios of (i17:0+i15:0)/(a17:0+a15:0) and cy17:0/16:1ω7c were used to ascertain nutritional or environmental stress [33], [37].

### Statistical Analysis

To explore variation in soil microbial community composition between treatments and among alpine grasslands, the mole percentages of individual PLFAs were subjected to principal component analysis (PCA) after standardizing to unit variance. PCA was carried out with the ‘rda’ function in the ‘vegan’ library using R statistical software (version 3.0.2) [38]. To assess the compositional dissimilarity of microbial communities between control and experimental warming, the Euclidean distance was calculated. The significance of the difference in the Euclidean distances was determined by permutation tests (999 permutations). The loading scores for individual PLFAs were used to assess their relative importance. Two-tailed paired t-tests were performed by the R program to calculate the significant differences of various variables between each warming and control treatment. Figures were generated by Sigmaplot 10.0 (Systat Software Inc.).

To explore the PLFA data explained by a linear model of environmental variables, redundancy analysis (RDA) was carried out with the ‘rda’ function in the ‘vegan’ library of the R program. The ordination of the response variables (PLFA data) was constrained by multiple regression analysis of the explanatory variables (environmental data). The significance of the RDA results was assessed by permutation tests (999 permutations). Because P<0.01, we presented the ordination biplot, which shows site scores as points and environmental variables as vectors. The angles in the biplot between response and explanatory variables, and between explanatory variables themselves, reflect their correlations. The proportion of explained variation was calculated by using adjusted R-squared values as described by Peres-Neto et al. [39]. The biplot was generated by the R program.

## Results

### Environmental Variables

Plant coverage was significantly different (P<0.05) between the warming and control plots of AM (31.7% and 24.5%, respectively), but was only slightly higher in the warming than the control plots of ASM (46.2 and 44.0%, respectively) and AS (21.2 and 19.9%, respectively). Three years of experimental warming increased the annual mean soil temperature by 1.7, 0.5, and 0.4°C in ASM, by 2.3, 1.5, and 1.0°C in AM, and by 1.7, 1.4, and 0.3°C in AS at the 0, 10, and 20 cm soil depths, respectively. Soil moisture was significantly decreased by warming at the 0–10 cm depth of ASM and at the 0–10 and 10–20 cm depths of AM and AS (P<0.05, Table 1). Soil moisture was significantly higher at the 10–20 cm depth regardless of grassland type and significantly lower in AS than ASM and AM (P<0.05, Table 1).

**Table 1:** Effect of experimental warming on selected soil properties at the 0–10 and 10–20 cm depths of alpine swamp meadow, alpine meadow, and alpine steppe.

	Soil moisture (%)	Soil organic C (g kg^−1^)	Total nitrogen (g kg^−1^)	C/N	pH
Alpine swamp meadow					
0–10 cm					
Control	39.3 (0.51) b†	22.4 (4.27) a	1.61 (0.21) a	13.7 (0.81) a	8.77 (0.04) a
Warming	37.2 (0.60) a	29.1 (6.21) b	1.96 (0.28) b	14.6 (1.01) b	8.60 (0.15) a
10–20 cm					
Control	46.8 (0.94) a	22.6 (1.64) a	1.53 (0.07) a	14.7 (0.44) a	8.76 (0.02) a
Warming	44.4 (0.80) a	25.3 (2.46) a	1.61 (0.14) a	15.6 (0.24) b	8.80 (0.03) a
Alpine meadow					
0–10 cm					
Control	37.8 (0.59) b	16.5 (0.76) a	1.25 (0.06) a	13.2 (0.23) a	8.69 (0.08) a
Warming	35.8 (0.80) a	17.1 (1.88) a	1.23 (0.15) a	14.0 (0.20) a	8.73 (0.16) a
10–20 cm					
Control	43.7 (0.83) b	13.5 (1.06) a	1.16 (0.04) b	11.6 (0.49) a	8.80 (0.06) a
Warming	41.9 (0.65) a	11.1 (0.99) a	0.98 (0.04) a	11.3 (0.49) a	8.80 (0.08) a
Alpine steppe					
0–10 cm					
Control	7.08 (0.13) b	4.97 (0.20) a	0.39 (0.07) a	13.6 (2.00) a	8.58 (0.04) a
Warming	6.16 (0.14) a	4.77 (0.46) a	0.40 (0.05) a	12.3 (1.09) a	8.59 (0.10) a
10–20 cm					
Control	8.89 (0.36) b	3.95 (0.56) a	0.35 (0.04) a	11.3 (0.41) a	8.57 (0.05) a
Warming	7.91 (0.24) a	4.31 (0.31) a	0.27 (0.01) a	15.9 (1.80) b	8.59 (0.07) a

† Values in the parentheses are standard errors. Different small-case letters between control and warming indicate significant differences at the 0.05 probability level (two-tailed paired t-test).

Soil organic C and total N levels were highest in ASM and lowest in AS and were generally less in the lower soil depths (Table 1). Experimental warming increased the soil organic C and total N levels by 30% and 22%, respectively, at the 0–10 cm depth of ASM, but decreased total N by 16% at the 10–20 cm depth of AM (P<0.05, Table 1). Soil C to N (C/N) ratios were significantly increased by warming at the 0–10 and 10–20 cm depths of ASM and at the 10–20 cm depth of AS (P<0.05, Table 1). All soils were slightly alkaline with pH values ranging from 8.57 to 8.80.

### Abundance of Soil Microbial Communities

**Figure 1:**
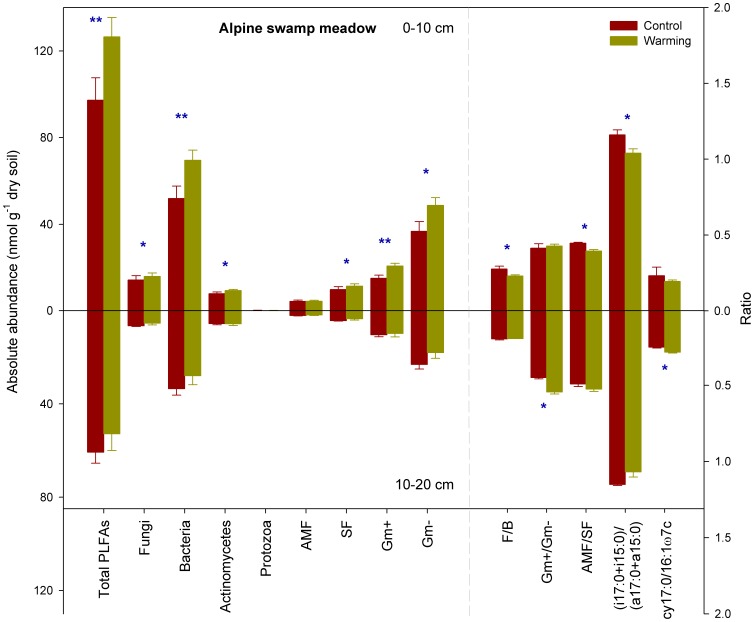
Sums and ratios of microbial groups in control and warming plots of alpine swamp meadow. Error bars indicate standard errors. Differences between the control and warming plots were analyzed by two-tailed paired t-tests, indicated by ** for P<0.01 and * for P<0.05. PLFA, phospholipid fatty acid; AMF, arbuscular mycorrhizal fungi; SF, saprotrophic fungi; Gm+, gram-positive bacteria; Gm-, gram-negative bacteria; F/B, the ratio of fungi to bacteria; Gm+/Gm-, the ratio of gram-positive to gram negative bacteria; AMF/SF, the ratio of arbuscular mycorrhizal fungi to saprotrophic fungi.

In the ASM ecosystem, experimental warming significantly influenced the total microbial biomass (total PLFAs) at the 0–10 cm soil depth (P<0.05, Fig. 1). The total microbial biomass was significantly higher in the warming versus the control plots at the 0–10 cm depth, due primarily to a significant increase in the abundance of all investigated microbial groups except protozoa and AMF (P<0.05, Fig. 1). The ratios of fungi to bacteria (F/B), arbuscular mycorrhizal to saprotrophic fungi (AMF/SF), and (i17:0+i15:0)/(a17:0+a15:0) ratios were significantly decreased as a result of warming at the 0–10 cm depth (P<0.05, Fig. 1). Total microbial biomass and the abundance of specific microbial groups were significantly decreased at the lower soil depths (P<0.05, Fig. 1). Warming did not influence the total microbial biomass or abundance of specific microbial groups at the 10–20 cm depth, but significantly increased the Gm+/Gm- and cy17:0/16:1ω9c ratios at this layer (Fig. 1).

**Figure 2:**
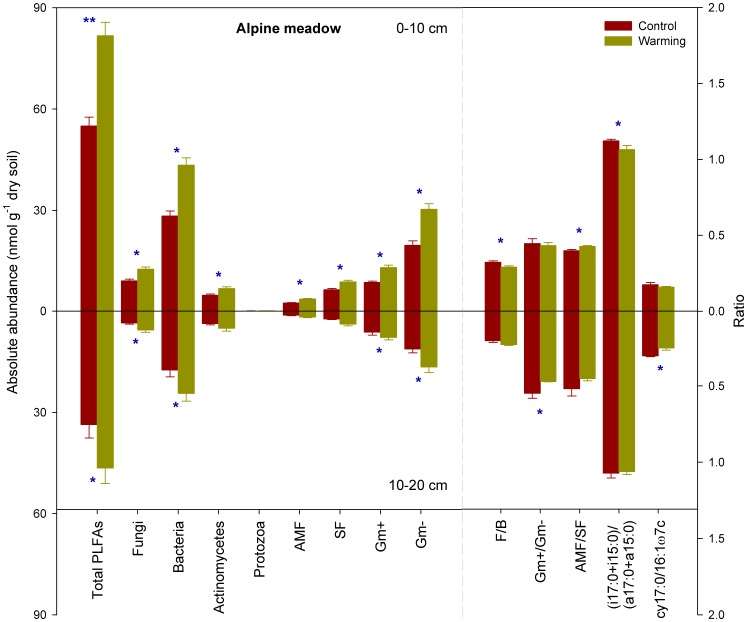
Sums and ratios of various microbial groups in control and warming plots of alpine meadow. Error bars indicate standard errors. Differences between the control and warming plots were analyzed by two-tailed paired t-tests, indicated by ** for P<0.01 and * for P<0.05. PLFA, phospholipid fatty acid; AMF, arbuscular mycorrhizal fungi; SF, saprotrophic fungi; Gm+, gram-positive bacteria; Gm-, gram-negative bacteria; F/B, the ratio of fungi to bacteria; Gm+/Gm-, the ratio of gram-positive to gram negative bacteria; AMF/SF, the ratio of arbuscular mycorrhizal to saprotrophic fungi.

The total microbial biomass and abundance of specific microbial groups in AM were significantly lower than those in ASM (P<0.05, Figs. 1 and 2). Experimental warming significantly increased the total microbial biomass and abundance of specific microbial groups except protozoa at the 0–10 cm soil depth of AM (P<0.05, Fig. 2). In addition, although F/B and (i17:0+i15:0)/(a17:0+a15:0) ratios were significantly lower, AMF/SF ratios were significantly higher in the warming versus control plots at this layer (P<0.05, Fig. 2). At the 10–20 cm depth of AM, the increase in total microbial biomass as a result of warming mainly stemmed from an increased abundance of fungi and Gm+ and Gm- bacteria (P<0.05, Fig. 2). In addition, experimental warming significantly decreased the Gm+/Gm- and cy17:0/16:1ω9c ratios at this depth (P<0.05, Fig. 2).

**Figure 3:**
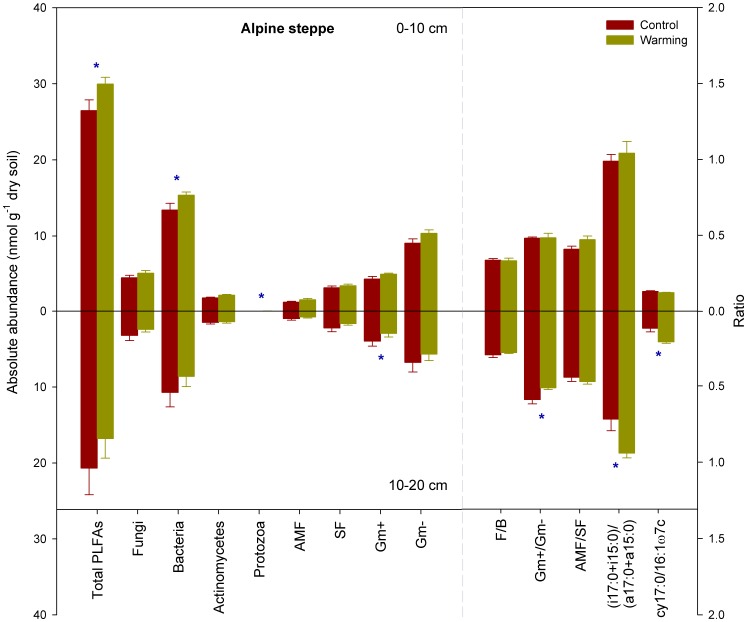
Sums and ratios of various microbial groups in control and warming plots of alpine steppe. Error bars indicate standard errors. Differences between the control and warming plots were analyzed by two-tailed paired t-tests, indicated by ** for P<0.01 and * for P<0.05. PLFA, phospholipid fatty acid; AMF, arbuscular mycorrhizal fungi; SF, saprotrophic fungi; Gm+, gram-positive bacteria; Gm-, gram-negative bacteria; F/B, the ratio of fungi to bacteria; Gm+/Gm-, the ratio of gram-positive to gram negative bacteria; AMF/SF, the ratio of arbuscular mycorrhizal fungi to saprotrophic fungi.

Compared to the soils of ASM and AM, those of AS contained the lowest total microbial biomass and abundance of specific microbial groups (Figs. 1, 2, and 3). Experimental warming of AS significantly increased bacteria and protozoa at the 0–10 cm soil depth but decreased the Gm+ bacteria at the 10–20 cm depth (P<0.05, Fig. 3). Furthermore, these warming plots had significantly higher cy17:0/16:1ω9c and (i17:0+i15:0)/(a17:0+a15:0) ratios but significantly lower Gm+/Gm- ratios at the 10–20 cm depth compared to the control plots (P<0.05, Fig. 3).

### Composition of Soil Microbial Communities

**Figure 4:**
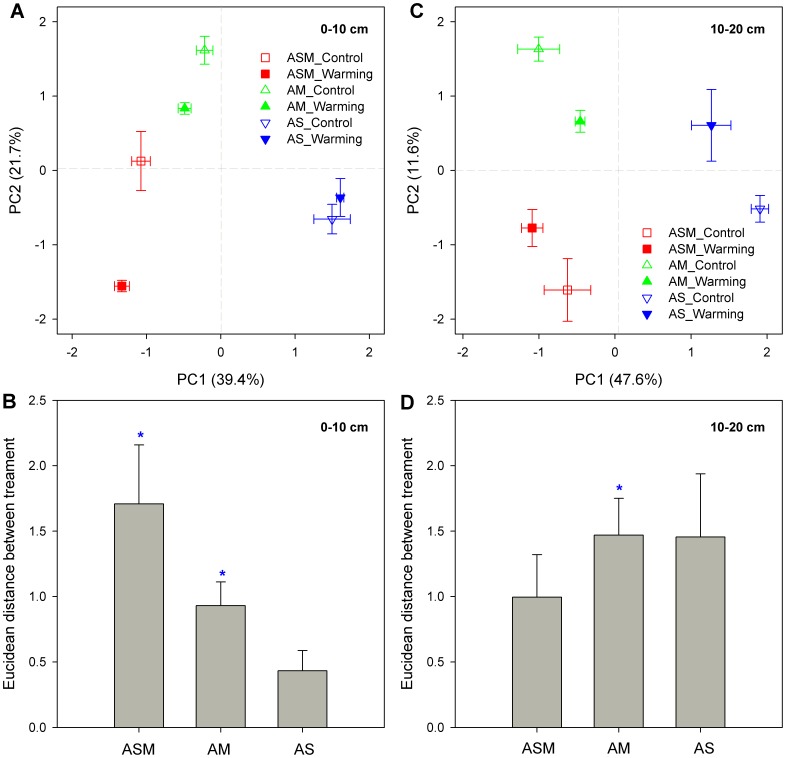
Principal component analysis (PCA) of the phospholipid fatty acid data in alpine grasslands. The composition of soil microbial communities (A and C) and the composition dissimilarity between the control and warming treatments (B and D) at the 0–10 and 10–20 cm soil depths of alpine swamp meadow (ASM), alpine meadow (AM), and alpine steppe (AS). Error bars indicate standard errors. Dissimilarity was assessed by calculating the Euclidean distances between the PC values. Star symbols indicate significant differences in the mean values of dissimilarity at the 0.05 probability level based on permutation tests (999 permutations).

At the 0–10 cm soil depth, the first principal component (PC1) and the second principal component (PC2) explained 39.4% and 21.7% of the total variance in the PLFA data, respectively (Fig. 4A). The ASM plots with the highest soil organic C levels are shown on the left-hand side of Figure 4A, the AM plots with an intermediate organic C levels in the middle portion of Figure 4A, and the AS plots with the lowest organic C levels are on the right-hand side of Figure 4A. Along the PC2 axis, the warming plots of ASM and AM scored higher than their corresponding control plots (Fig. 4A). The PCA plot also showed that data points for AS were intermixed. The Euclidean distance of soil microbial community composition between control and warming was significant in the ASM and AM ecosystems at this layer (P<0.05, Fig. 4B). The loading scores for individual PLFAs revealed 10Me17:0, 17:1ω8c, 18:1ω9c, and cy19:0 were most important for the separation of sites along the PC1 (Table S1).

At the 10–20 cm soil depth, the PC1 and PC2 explained, respectively, 47.6 and 11.6% of the total variance in the PLFA data (Fig. 4C). The data points for ASM and AM were on the left-hand side and those for AS on the right-hand side (Fig. 4C). Permutation tests suggested significant differences in soil microbial community composition between control and warming plots in the AM ecosystem at this layer (P<0.05, Fig. 4D). Lipid signatures 16:1ω5c, 17:1ω8c, and 18:1ω9c had higher positive loading scores while cy19:0 and 20:0 had lower negative loading scores along the PC1 axis (Table S1).

### Relationship between PLFA and Environmental Data

**Figure 5:**
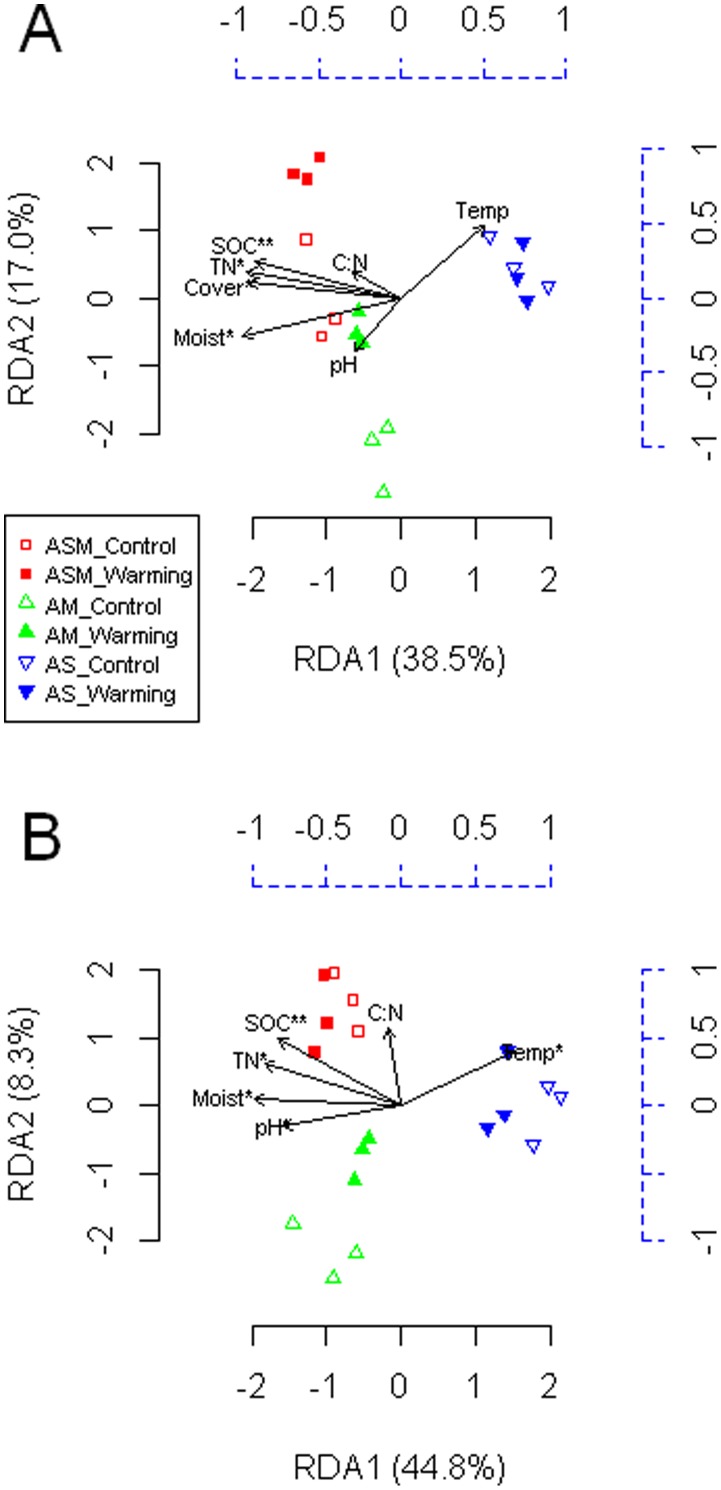
Redundancy analysis (RDA) of the phospholipid fatty acid (PLFA) data as explained by environmental variables. Seven and six environmental variables were used for the 0–10 (A) and 10–20 cm (B) soil depths, respectively. The PLFA data are scaled by the black solid axes (bottom and left), and the explanatory variables are scaled by the blue dashed axes (top and right). The proportion of explained variation was calculated by using adjusted R-squared values as described by Peres-Neto et al. (2006). The explanatory variables followed by an asterisk indicate significant influences on the PLFA data (*, P<0.05; **, P<0.01). ASM, alpine swamp meadow; AM, alpine meadow; AS, alpine steppe; SOC, soil organic C; TN, total nitrogen; Moist, soil moisture; Temp, soil temperature; Cover, plant coverage.

Redundancy analysis showed that the first and second canonical axes explained 38.5 and 17.0%, respectively, of the total variance in the PLFA data at the 0–10 cm depth, and 44.8 and 8.3%, respectively, of the total variance at the 10–20 cm depth (Fig. 5). Because ecological data are generally quite noisy, we are confident that the major trends have been modeled in this analysis. Furthermore, the first unconstrained eigenvalues are comparatively small, which means that they do not display any important residual structure of the PLFA data. Soil organic C, total N, soil moisture, and plant coverage had a significant effect on soil microbial communities at the 0–10 cm depth (P<0.05, Fig. 5A). For the 10–20 cm depth, organic C, total N, soil moisture, pH, and soil temperature were important environmental factors (P<0.05, Fig. 5B).

## Discussion

### Warming Effect on the Abundance of Soil Microbial Communities

Our results clearly show that 3 years of experimental warming consistently and significantly increased the abundance of microbial communities at the 0–10 cm soil depth of all studied alpine grasslands. These results align well with studies showing significant increases in microbial biomass in a range of Antarctic environments after 3 years of passive warming [9], but differ from the common finding of most warming studies that microbial biomass either remained at steady levels or decreased depending on the duration of warming [12]–[15], [40], [41]. These disparities most likely stem from the prevailing cold climate conditions in our study and that of Yergeau et al. [9]. Soil microorganisms in cold environments have optimal growing temperatures far above field conditions [19], [42], which potentially enable them to increase their biomasses rapidly under elevated temperatures [43]. Therefore, temperature had a direct effect on microbial biomass. However, it seems that the direct effect of warming on microbial biomass accumulation was significant only when the soil temperature increase reaches a certain value. When we averaged the soil temperatures at 0 and 10 cm for the 0–10 cm soil depth and at 10 and 20 cm for the 10–20 cm soil depth, we obtained increases in soil temperature of 1.10, 1.90, and 1.55°C at the 0–10 cm soil depth and 0.45, 1.25, and 0.85°C at the 10–20 cm soil depth of ASM, AM, and AS, respectively. Given that there were generally no significant differences in microbial biomass between control and warming plots at the 10–20 cm soil depth of ASM and AS, these averaged temperature values suggest that microbial biomass did not accumulate when the temperature increased ≤0.85°C. Further research is needed to elucidate this point.

In addition to a direct temperature effect, warming may indirectly affect soil microbial abundance by influencing aboveground biomass [40]. In this study, warming increased total plant coverage by 4.9, 29.3, and 6.1% in ASM, AM, and AS, respectively. Increased aboveground biomass may introduce more organic C into soils (through plant litters and root exudates), which may have a strong influence on substrate availability and consequently on the growth and activity of soil microbial communities [44]. The warming effect on plant coverage was so profound in the AM grasslands that the increased organic C input may have provided an ample supply of substrates even at the 10–20 cm soil depth, and thus partially contributed to the significant increase in soil microbial biomass at this layer. Higher microbial biomass is usually accompanied by higher heterotrophic respiration [10]. It seems that the increase in C loss through soil respiration was offset by the increase in C inputs from aboveground biomass at the 0–10 cm depth of AM and AS and at the 10–20 cm depth of AM. As a result, total organic C remained unchanged at these soil layers. However, warming significantly increased total organic C at the 0–10 cm depth of ASM, suggesting a negative feedback between soil microbial communities and C storage under experimental warming. These data highlight the importance of conserving the ASM ecosystem as a C sink to mitigate climate change in the studied region.

Soil moisture has considerable influence over soil microorganisms [45]. In this study, experimental warming significantly decreased soil moisture at the 0–10 cm depth of ASM and AM and at the 10–20 cm depth of AM. Because soil moisture is relatively high in ASM and AM ecosystems, a decrease in soil moisture most likely indicates reduced physiological stress, which might partially contribute to the observed increase in soil microbial biomass at these layers [18], [46]. This is supported by significantly lower stress indicators at the 0–10 cm depth of warmed plots in ASM and AM ((i17:0+i15:0)/(a17:0+a15:0)) and at the 10–20 cm depth of warmed plots in AM (cy17:0/16:1ω9c).

### Warming Effect on Composition of Soil Microbial Communities

Warming has been reported to markedly shift the composition of soil microbial communities across a wide range of ecosystems [4], [9], [15], [16]. The results from our study also showed that the microbial composition had changed significantly following 3 years of experimental warming at the 0–10 cm depth of ASM and AM and at the 10–20 cm depth of AM, as indicated by PCA-based ordination of the PLFA data. These data suggest that the warming effects on soil microbial composition were dependent on grassland type and soil depth. RDA revealed that microbial composition at the 0–10 cm soil depth was primarily controlled by plant coverage, soil organic C, total N, and soil moisture, suggesting that the warming effect on soil microbial communities at this layer was indirect and mainly influenced by vegetation inputs and the microbial habitat of our study area. Alternatively, temperature effects may not be explained by a simple linear relationship. RDA also indicated that the composition of the soil microbial community at the 10–20 cm depth was heavily shaped by environmental factors.

Our results indicated a community shift towards bacteria at the surface layer of the warming plots, which is consistent with a significant reduction in the relative abundance of fungi after warming in a subarctic heath ecosystem [14] and within forest soil [13]. Competition for substrates between microorganisms likely explains this observation. Increased substrate availability and decreased environmental stress under warming likely give bacteria a competitive advantage over fungi with regards to available C and other nutrients [47]. Consequently, bacteria would grow faster than fungi, leading to the observed bacterial dominance in the soil microbial community at the 0–10 cm depth of ASM and AM ecosystems.

Another composition change in the warmed soil microbial communities was the relative abundance of AMF and SF at the 0–10 cm depth of ASM and AM. Saprotrophic fungi contribute to decomposition mostly through enzymatic action, promoting cell lysis and degrading cell constituents [48]. Higher microbial biomass is generally accompanied by higher production of microbial necromass [49], which most likely provides substrates for SF and thus contributed to greater SF abundance in warming plots of ASM and AM. However, AMF was not increased by warming in ASM due to their weak saprotrophic capacity [43], which led to significantly lower AMF/SF ratios in warming versus control plots. The significantly higher AMF/SF ratio in warming plots of AM suggests a greater increase in the abundance of AMF. Increased AMF most likely resulted from their association with plant roots, which increased due to significantly higher plant coverage [50], [51]. Thus, plant coverage plays an important role in influencing the relative abundance of AMF and SF at the 0–10 cm soil depth of ASM and AM.

## Conclusions

Results from our study indicate that 3 years of experimental warming leads to significant increases in microbial biomass of alpine grasslands, due primarily to changes in soil temperature, moisture, and plant coverage. However, soil organic C content did not decrease as a result of increased microbial biomass in alpine meadow and alpine steppe, suggesting that increased C loss through soil respiration was offset by an increase in C inputs from plants. The significantly higher soil organic C in warming plots of alpine swamp meadow highlights the importance of conserving this type of ecosystem on the Qinghai-Tibet Plateau. Warming also significantly decreased the fungal to bacterial ratio and induced changes in the relative abundance of arbuscular mycorrhizal and saprotrophic fungi at the 0–10 cm depth of ASM and AM. These changes mainly resulted from variations in the aboveground plant coverage and soil environmental conditions. Conversely, soil microbial community composition at the 10–20 cm depth was primarily influenced by environmental variables. Our results suggest that in response to climate warming, microorganisms within the soil may have a profound impact over long-term ecosystem feedback loops.

## Supporting Information

Table S1 Top 5 phospholipid fatty acids (PLFAs) most responsible for the changes in lipid signatures along the first principal component (PC1).
(DOC)
