# Supplementary material for: Responses of Soil Microbial Communities to Experimental Warming in Alpine Grasslands on the Qinghai-Tibet Plateau
Source: PLoS One. 2014 Aug 1;9(8):e103859. doi: 10.1371/journal.pone.0103859 (PMC4118913; doi:10.1371/journal.pone.0103859)
Supplement: Table S1 — Top 5 phospholipid fatty acids (PLFAs) most responsible for the changes in lipid signatures along the first principal component (PC1). (DOC) [file pone.0103859.s001.doc]

**Table S1**

| PLFAs | Species loading scores | |
| --- | --- | --- |
|  | 0–10 cm | 10–20 cm |
| 16:1ω5c |  | 0.765 |
| 17:1ω8c | 0.789 | 0.768 |
| 10Me17:0 | 0.712 |  |
| 18:1ω9c | 0.726 | 0.766 |
| cy19:0 | -0.752 | -0.805 |
| 20:0 | -0.775 | -0.775 |
